# Clinical outcomes of Sodium-glucose cotransporter-2 inhibitors in patients with Type 2 Diabetes Mellitus: An observational study from Pakistan

**DOI:** 10.12669/pjms.37.5.3901

**Published:** 2021

**Authors:** Bhagwan Das, Aisha Sheikh, Bilal Ahmed, Najmul Islam

**Affiliations:** 1 Bhagwan Das, FCPS. Department of Medicine, Section of Endocrinology, Aga Khan University, Karachi, Pakistan; 2 Aisha Sheikh, FCPS. Department of Medicine, Section of Endocrinology, Aga Khan University, Karachi, Pakistan; 3 Bilal Ahmed, PhD. Department of Medicine, Aga Khan University, Karachi, Pakistan; 4 Najmul Islam, FRCP. Department of Medicine, Section of Endocrinology, Aga Khan University, Karachi, Pakistan

**Keywords:** Glycated Hemoglobin A, Hypoglycemic Agents, Sodium-Glucose Transporter 2 Inhibitors, Diabetes Mellitus, Type-2

## Abstract

**Objective:**

To determine the efficacy and safety of Sodium-glucose cotransporter-2 inhibitors (SGLT2i) use in the Pakistani population.

**Methods:**

Retrospective review of initial 100 patients who were prescribed with any agent of the SGLT2i group from July 1, 2018, to January 2019 at Aga Khan University Hospital, Karachi. SGLT2i was offered to patients of above 18 years of age with inadequate glycemic control on existing antidiabetic agents. Changes in HbA1c, the Body Mass Index (BMI), serum creatinine, any decrease in the requirement of insulin and sulphonylurea dose along with any side effects reported by the patients on follow-up visits.

**Results:**

Most study participants were females (56%) with the mean age of 52±10 years. Substantial changes were observed in the HbA1c (7.5±1.1%, 7.9±1.2% from 8.7±1.5%, p < 0.01), BMI (31.4±5.8, 31.8±5.8 from 32.4±5.9kg/m^2^, p < 0.01) and in creatinine (0.71±0.1, 0.75±0.1 from 0.79±0.1 mg/dl, p < 0.01) at three and six months of follow up visits. The reduction in insulin and sulphonylurea doses was also significant. Adverse drug events that led to drug discontinuation in 14 individuals were, Urinary tract infection (UTI) (seven patients), Genital infection (three patients), nausea +UTI, abdominal pain +UTI, mild Diabetic Ketoacidosis, and polyuria (one patient each). None reported Fournier’s gangrene, limb amputation, or fracture.

**Conclusion:**

SGLT2i significantly improved glycemic control, BMI, and serum creatinine in the Pakistani population with a very low number of observed adverse events.

## INTRODUCTION

Diabetes Mellitus is a serious public health concern with a worldwide estimated prevalence of 460 million in the adult population of age between 20 to 79 years and 79.4 % of this estimation is living in low to middle-income countries including Pakistan^1^. In Pakistan estimated prevalence of diabetes mellitus is about 17.1%^1^ with compliance of only 60 % with prescribed anti diabetic medications.^2^ Diabetes mellitus Type-2 (T2DM) is a slowly progressive metabolic disorder characterized by insulin resistance and a progressive defect in insulin secretion associated with severe macrovascular and microvascular complications.^3^ Because of its progressive nature, it needs multiple anti-diabetic agents for achieving and maintaining optimum glycemic control, unfortunately, more than 50 to 70% of patients with T2DM failed to achieve and maintain their glycemic control at some stage.^4^,^5^ Sodium-glucose co-transporter 2 inhibitors (SGLT2i) are the new oral agents for the management of T2DM that can be used as an add on therapy with other oral agents and insulin or as a monotherapy at any stage of diabetes. These novel agents improve glycemic control by inhibiting glucose reabsorption through SGLT2 transmembrane proteins at the level of proximal renal tubules and re-entering circulation.^6^ Selective inhibition of SGLT2 has shown a potent antihyperglycemic effect with additional favorable effects such as reduction in the weight and blood pressure.^7^

Since the results of large trials on this group, empagliflozin cardiovascular outcome event trial (EMPA-REG OUTCOME), dapagliflozin effect on cardiovascular events–thrombolysis in myocardial infarction58( DECLARE-TIMI58) and canagliflozin cardiovascular assessment study (CANVAS), it has been shown that these newer oral anti-diabetic agents reduce the incidence of cardiovascular events in patients with T2DM and established cardiovascular disease^8^-^10^, there has been great interest of endocrinologists and cardiologists in expanding use of this group of drug in T2DM patients with cardiovascular diseases, especially heart failure.^11^

Empagliflozin and dapagliflozin are the two FDA approved drugs of this SGLT2i group, currently available in Pakistan. These both are the recommended options for the management of T2DM either as a monotherapy or as add-on therapy with other antidiabetic agents^12^ with a reported improvement in HbA1c up to 1.8%.^13^ The most common adverse effects reported with these novel agents are genital infections (GIs) and urinary tract infections (UTIs).

There is limited published data on the efficacy and safety of both agents of this novel anti-diabetic group, empagliflozin and dapagliflozin, among the Pakistani population.^14^, ^15^ Hence, we aimed this study to know about the efficacy of SGLT2i in terms of glycemic control and to report its effects on other metabolic parameters like reduction in the Body Mass Index (BMI) and change in the serum creatinine level along with adverse effects on Pakistani population in real-world practice.

## METHODS

This retrospective cohort study was conducted at the outpatient clinics of the Endocrine section of Aga Khan University Hospital Karachi (AKUH), Pakistan. The Endocrine clinics of our hospital cater more than 170 patients daily out of which more than 90% are having T2DM.

We reviewed the outpatient records of all the T2DM patients who were prescribed with any agent of the SGLT2 inhibitors group (dapagliflozin or empagliflozin) from 1st July 2018 to 31^st^ January 2019, due to failure to achieve adequate glycemic control on their existing antidiabetic agents. The protocol of this study was approved by the university’s ethical committee (ERC number 2019-1034-2727, dated February 19, 2019) and the study was carried out under the Helsinki ethics principles. To preserve the confidentiality of study participants, we coded each participant and removed their personal details.

Data collection was started by reviewing the files at Health Information Management System (HIMS) of AKUH and was noted in the predefined study questionnaire by the primary author. We recorded the data regarding patients’ demographics including age, gender, antidiabetic agents they were taking before starting SGLT2i, baseline HbA1c, BMI, and serum creatinine level.

### Inclusion and Exclusion criteria

We included the patients who were of 18 years or older,of either gender with insufficient glycemic control (HbA1c > 7%). We excluded all patients with T1DM, pregnant women, or those planning to conceive, history of recurrent UTI (with a frequency of at least three UTIs/year or two UTIs in the last six months), renal impairment, any malignancy, chronic liver disease, on any medicine like steroids, antibiotics, current or previous acute complications of diabetes like hyperglycemic hyperosmolar state, diabetic ketoacidosis (DKA), or any electrolyte imbalance.

The primary endpoint for our study was the change in the HbA1c at three and six months from baseline. Secondary endpoints were the possible changes in the BMI, daily insulin and or sulphonylureas (SU) dose, and serum creatinine from baseline at three and six months of follow up OPD visits. Safety was also assessed via reporting of adverse drug events (AEs) on the basis of history and lab evidence where appropriate. AEs of special interest, UTI, GTI, volume depletion/hypotension, diabetic ketoacidosis (DKA), electrolyte imbalance, gangrene, spontaneous fracture, or any other noted by the study participant.

### Statistical analysis

Mean ± standard deviation (SD) was calculated for the continuous variables, percentages, and counts were calculated for the nominal variables. To determine the changes in baseline clinical characteristics according to the improvement in HbA1c, independent t-test was used for continuous variables and Pearson’s chi-square test was used for the categorical variables. We compared HbA1c, BMI at baseline, and at three and six months using the paired t-test considering p-value of <0.001 as statistically significant at a confidence interval of 95%. All the statistical analysis was performed on SPSS version 19.0 (IBM Co., USA).

## RESULTS

Among the Type-2 diabetic patients, initial 100 patients who were prescribed with SGLT2 inhibitors and who followed us on 3 and 6 months as an outpatient were included in this study. The mean age of the study participants was 52 years and the majority (56%) were females. The average duration of Diabetes was 12 years and the mean BMI was 32.4 ±5.9kg/m^2^. About half (49%) of the participants were insulin users and one-third (32%) were on SU. Table-I.

**Table-I:** Baseline clinical characteristics of the study participants.

Age (years)	51.9 ±10.4
Gender n (%)	
Male	44 (44)
Female	56 (56)
BMI, kg/m^2^	32.4 ±5.9
Creatinine	0.8 ±0.1
Current treatment	
Insulin takers (BB + premix)	49 (49%)
SU	32 (32%)
SU + Basal Insulin	9 (9%)
Others	10 (10%)

BB: Basal Bolus, SU: Sulphonylureas.

After three months of SGLT2 intake, HbA1C levels substantially improved from a baseline value of 8.7 ± 1.5% to 7.9 ± 1.2% (P < 0.01). Likewise, BMI decreased from 32.4±5.9kg/m^2^. kg/m^2^ at baseline to 31.8±5.8 kg/m2 (P < 0.01) at 3-months. After six months of SGLT2 treatment, HbA1C levels further decreased at 7.5 ± 1.1 in comparison to baseline measurements (P < 0.01). Similarly, the BMI of the patients improved from 32.4±5.9kg/m^2^. kg/m^2^ at baseline to 31.4±5.8kg/m^2^ at 6 months of treatment. The average insulin requirement has decreased from 93.5±55.8 IU at baseline to 85±49.3 IU at three months and then 81.4±47.7 IU at six months (P < 0.01). Table-II and III.

**Table-II:** Endpoints with changes at three and six months.

Variable	Baseline	3 months	6 months	p-value *
HbA1C	8.7 ± 1.5	7.9 ± 1.2	7.5 ±1.1	< 0.01
BMI, kg/m^2^	32.4±5.9	31.8±5.8	31.4±5.8	< 0.01
Creatinine	0.79 ±0.1	0.75 ±0.1	0.71 ±0.1	< 0.01
Medications				
SU				
Glimepiride (mg/day)	5.1 ±2.4	4.2 ±2.8	3.9 ±2.9	0.01
Gliclizides (mg/day)	75 ±37.9	58 ±11.1	52.5 ±43.1	0.01
Insulin (units/day)				
Insulin	93.5 ±55.8	85 ±49.3	81.4 ±47.7	< 0.01

Baseline was taken as Reference.

**Table-III:** Adverse Drug events observed with SGLT2i.

ADE types	Percentage
None	86%
UTI	7%
Genital inflammation	3%
DKA	1%
Nausea and UTI	1%
Abdominal pain and UTI	1%
Polyuria	1%

## DISCUSSION

This single-center 6 months study from Pakistan shows SGLT2 inhibitors are effective in the Pakistani population not only in glycemic control but also in the weight reduction and other metabolic effects with fewer side effects. Our study results further strengthens the outcome data from the previous studies,^7^,^8^,^16^ mostly done in the developed countries, by showing significant improvement in different metabolic parameters in the diverse population with T2DM in the real-world setting in Pakistan.

Reduction in the HbA1c observed in this study, from 8.7 ± 1.5% to 7.9 ± 1.2% at 3 months and further reduction to 7.5 ± 1.1 at 6 months is comparable to the results of other real world studies and trials in which agents of this group were either used in combination or as monotherapy and showed improvement in HbA1c up to 1.8%.^13^-^18^ This reduction in HbA1c and improvement in insulin sensitivity and decrease in insulin resistance along with a reduction in the beta-cell loss as reported by Merocvi A, et al.^19^ make SGLT2i as a potent agent to be used in combination or as monotherapy as supported by EASD- ADA position statement for DM management.^20^

Most patients with T2DM are obese or overweight and weight loss in a diabetic patient is among the desired outcome because of its impact on insulin sensitivity, overall glycemic control, and long-term morbidities.^21^ A decrease in the BMI noted in our study is also comparable to other studies.^16^,^18^ We found a decrease in BMI from 32.4 ±5.9 kg/m^2^ to 31.8±5.8 kg/m^2^ and 31.4±5.8 kg/m^2^ at 3 and 6 months respectively. Weight reduction associated with SGLT2i likely observed is mainly because of their glycosuria induced energy loss and reduction in the fat mass was found to be sustained for more than two years of treatment as reported by Bolinder J et al.^22^ Because of this weight reduction and improvement in BMI one can expect a decrease in daily dose requirement of insulin and SU as seen in this study and other clinical studies on this drug group.^16^, ^18^, ^23^ This decrease in insulin and SU further decreases the chances of weight gain and the risk of hypoglycemia associated with both of these agents.

We observed a decrease in serum creatinine from 0.79±0.1 mg/dl to 0.71±0.1mg/dl and 0.75±0.1mg/dl at 3 and 6 months. Evidence from different trials, meta-analysis, systemic reviews, and real-world studies has shown the renoprotective effect of SGLT2Is, independent of their glycemic effects.^24^-^26^ They have been associated with a reduction in the risk for major adverse renal outcomes among T2DM patients and have also shown a decrease in the progression of renal disease over a follow-up period of 3.1 years.^9^ CANVAS program also reported a reduction in the multiple renal outcomes up to 40%, including improvement in the estimated glomerular filtration rate, eGFR and in the progression of proteinuria, need for renal replacement therapy, or mortality from renal causes.^24^

Treatment with SGLT2i in our study was reasonably well tolerated; most common AEs reported were urinary tract infections (7%) and genital tract infections (3%), no events were severe, were treated effectively along with discontinuation of the drug. Incidence of UTIs reported in the published clinical trials is also in a similar range, from 4% to 9 % and the raw event rate of GTIs has been reported around 4.7% in one recent meta-analysis by Liu et al.^27^ Given that SGLT2i are reported to be associated with an enhanced risk of UTI and GTI, further long term follow up studies are warranted to confirm these findings.

### Strengths & Limitations of the study

This is among the first few real-world studies on the efficacy and safety of SGLT2i from Pakistan with promising results, showing this group of the agent can be prescribed with confidence in our population. Being a retrospective study, it has some limitations in the collection of data, and, also being a single-center study with a small sample size, therefore, is not a true representation of the whole Pakistani population.

## CONCLUSION

Use of SGLT2i as add on therapy is effective in the Pakistani population not only in improving glycemic control but also leading to weight reduction and with a decrease in daily doses of sulphonylurea and insulin with a very low number of observed adverse events.

### Recommendation

Further prospective studies are needed in this part of the world to report their effects on cardiovascular, renal, and other chronic complications of diabetes.

### Authors’ Contribution:

**BD**: Concept, design, literature search, drafting, and approval of the final manuscript. Responsible and accountable for the integrity of the work.

**AS**: Concept, design, literature search, revised critically, patient management and approval of the final manuscript.

**BA**: Interpretation of data, preparation and approval of the final manuscript.

**NI**: Concept, design, supervision, patient management and approval of the final manuscript.
